# Notes

**Published:** 1902-01

**Authors:** 


### NOTES.

The National Live-stock Association held a meeting in the Stuttgart Theatre, Chicago, from the 3d to the 7th of December. A good many veterinarians were drawn to Chicago by this meeting and by the National Live-stock Exposition, which was held in Chicago during the same week.

Among the veterinarians present were Dr. W. H. Dalrymple, of Louisiana; Dr. J. O. Greeson, of Indiana; Dr. A. W. Bitting, of Indiana; Dr. M. E. Knowles, of Montana; Dr. Leonard Pearson, of Pennsylvania; Dr. C. A. Cary, of Alabama; Dr. A. T. Peters, of Nebraska; Dr. S. D. Brimhall, of Minnesota; Dr. J. J. Repp, Dr. J. H. McNeal, and Dr. L. A. Kline, of Ames, Iowa; Dr. J. I. Gibson, of Iowa; Dr. C. A. Gresswell, of Colorado.

The exposition consisted chiefly in a show of fat cattle and cattle of the beef breeds, especially the Aberdeen-Angus, Hereford, and Short-horns. It was the largest collection of cattle of this class that has ever been brought together in the United States. There were over 1000 pure-bred cattle in the show and more than 300 car-loads of fat cattle and breeders. There was also a very good exhibition of draught horses, sheep, and swine.

It is needless to say that the Chicago veterinarians were out in force. Drs. Pierce, Lovejoy, Hughes, McKillip, Baker, and Merillat were assiduous in their attendance, and the inspectors of the Bureau of Animal Industry, Drs. Dyson and Houck, were present as much as their other duties would permit.

The National Live-stock Association is composed chiefly of range cattlemen, and it is an organization in the interest of the range cattle industry, although there were a few delegates from the Eastern and Central States and from organizations not associated with the range cattle business. Of course, it was to be expected that such an organization would be opposed to the Grout bill, which proposed to put a tax on oleomargarine when it is colored to imitate butter. In fact, the organization has on every possible occasion taken opportunity to oppose the Grout bill.

When a resolution condemning this bill came before the organization a lively battle was waged. The opposing forces in this contest happened to be led by two veterinarians. Dr. Leonard Pearson, of Pennsylvania, was the spokesman for the side favoring the Grout bill, and Dr. M. E. Knowles, of Montana, was the floor manager and parliamentarian of the side opposed to the Grout bill. As was expected, the opponents of the bill carried their resolution, but by a majority much smaller than had been anticipated.

It was shown at this meeting that the live-stock industry of the country is in a most healthy and flourishing condition. There were never before as many animals in the country, and their value was never before as great.

Perhaps the most interesting address of the whole meeting was read by Mr. Le Grande Powers, in charge of the live-stock statistics of the United States census. Mr. Powers showed that as a result of a more accurate inquiry in relation to the number and distribution of farm animals made by the enumerators of the last census it has been found that there are far more domestic animals in the country than has hitherto been known.

Altogether the meeting was very successful, and, to the delegates from the West, profitable.

The horse-meat butcher shops of Vienna, of which there are no fewer than 185, present a clean and attractive appearance, and are in no way distinguishable from shops where the usual kinds of meat are sold save by the sign announcing their specialty. Restaurant keepers who serve horse-meat must designate this fact in a special column on the bill of fare offered to patrons.

The State of Ohio has a very active organization throughout its Commonwealth looking to the prevention and control of tuberculosis. Active representatives of the medical and veterinary professions, county and city boards of health, and others interested in public sanitation make up the body.

Mr. Carnegie's munificence: We have just learned from Professor McCormack, Secretary to the Carnegie Trust for the Universities of Scotland, that if a youth have the following qualifications his college (veterinary) fees will be paid by the Trust for him:

(1) He must be over sixteen years of age; (2) must be of Scottish birth or extraction, or must have given two years' attendance, after the age of fourteen years, at a school or institution under inspection of the Scotch Education Department; and (3) must be qualified by preliminary examinations under the ordinances of the Scottish Universities Commission and the regulations of the Joint Board of Examiners to attend the classes for which payment of fees has been claimed.—*Veterinary Journal*, December, 1901.

